# The necessity of IIb dissection in T1–T2N0M0 oral squamous cell carcinoma: protocol for a randomized controlled trial

**DOI:** 10.1186/s13063-019-3683-y

**Published:** 2019-10-22

**Authors:** Lei Wang, Liang Wang, Xuefei Song, Chang Cui, Chunyue Ma, Bing Guo, Xingjun Qin

**Affiliations:** 1 Department of Oral & Maxillofacial - Head & Neck Oncology, Shanghai Ninth People’s Hospital, College of Stomatology, Shanghai Jiao Tong University School of Medicine; National Clinical Research Center for Oral Diseases; Shanghai Key Laboratory of Stomatology & Shanghai Research Institute of Stomatology, 639, Zhi Zao Ju Road, Shanghai, 200011 China; 2 Department of Ophthalmology, Ninth People’s Hospital, Shanghai Jiao Tong University School of Medicine, Shanghai Key Laboratory of Orbital Diseases and Ocular Oncology, 639, Zhi Zao Ju Road, Shanghai, 200011 China

**Keywords:** Level IIb, Overall survival, T1–2N0M0 oral squamous cell carcinoma, Health-related quality of life, Randomized controlled trial

## Abstract

**Background:**

There is a growing debate on the relationship between health-related quality of life (HRQoL) and patient survival which has been going on for the last few decades. The greatest wish of clinicians is to extend the latter while improving the former. Following neck dissection of early-stage oral carcinoma, “shoulder syndrome” appears due to traction of the accessory nerve during removal of level IIb, which greatly affects patient quality of life. Since occult metastasis in level IIb of early-stage oral carcinoma is extremely low, some surgeons suggest that level IIb can be exempt from dissection to improve the HRQoL. However, other surgeons take the opposite view, and thus there is no consensus on the necessity of IIb dissection in T1–2N0M0 oral squamous cell carcinoma (OSCC).

**Methods:**

We designed a parallel-group, randomized, non-inferiority trial that is supported by Shanghai Ninth People’s Hospital, Shanghai Jiao Tong University, School of Medicine, Shanghai, China. We will enroll 522 patients with early oral carcinoma who match the inclusion criteria, and compare differences in 3-year overall survival, progression–free survival (PFS) and HRQoL under different interventions (retention or dissection of level IIb). The primary endpoints will be tested by means of two-sided log-rank tests. Analysis of overall and progression-free survival will be performed in subgroups that were defined according to stratification factors with the use of univariate Cox analysis. In addition, we will use post-hoc subgroup analyses on the basis of histological factors that were known to have effects on survival, such as death of invasion of the primary tumor. To evaluate HRQoL, we will choose the Constant–Murley scale to measure shoulder function.

**Discussion:**

Currently, there are no randomized controlled trials with large sample sizes on the necessity of IIB dissection in T1–T2N0M0 OSCC. We designed this noninferiority RCT that combines survival rate and HRQoL to assess the feasibility of IIb neck dissection. The result of this trial may guide clinical practice and change the criteria of how early-stage oral cancer is managed. The balance between survival and HRQoL in this trial is based on early-stage breast cancer treatment and may provide new ideas for other malignancies.

**Trial registration:**

Chinese Clinical Trial Registry, ChiCTR1800019128. Registered on 26 October 2018.

**Electronic supplementary material:**

The online version of this article (10.1186/s13063-019-3683-y) contains supplementary material, which is available to authorized users.

## Background

The surgical treatment of early-stage oral squamous cell carcinoma (OSCC) has been a dilemma for decades. In 2015, Anil K. D’Cruz published a randomized trial on the relationship between overall survival (OS) and the quality of life in patients who underwent two types of surgery for early-stage OSCC. The study demonstrated that the 3-year OS was greatly improved when patients underwent neck dissection and primary resection at the same time, compared with those who underwent primary resection but not neck dissection [1]. However, if we only pursue a higher survival rate, most patients will be regarded as ‘overtreated’. At present, the technique for detecting occult metastatic lymph nodes in the neck is still imprecise; we can detect susceptible nodes only by physical examination, B-scan ultrasonography, and enhanced computed tomography (CT)/magnetic resonance imaging (MRI), but diagnoses obtained by these methods are far from precise [2, 3]. Studies have shown that the rate of occult metastasis in the early stages of oral cancer is about 30% [1, 4, 5], meaning that nearly 70% of patients with negative nodes underwent neck dissection during routine treatment.

At present, we aim to extend survival and at the same time improve health-related quality of life (HRQoL) [6, 7]. After neck dissection, shoulder weaknesses such as dyskinesia, trapezius atrophy, loss of shoulder abduction, and shoulder and neck pain will occur; collectively, these are called ‘shoulder syndrome’ [8–10]. The physiological mechanism of this symptom is still unclear, but it can be explained anatomically. The accessory nerve innervates the sternocleidomastoid and trapezius muscles [11]; once the nerve is pulled during surgery, the function of both muscles is affected, and symptoms correspondingly occur. In addition, direct traction during surgery of the sternocleidomastoid muscle and other muscles associated with shoulder movement can damage the muscle bundle [10]. Therefore, shoulder syndrome can greatly affect postsurgical HRQoL. If the accessory nerve can be protected or the pulling of it avoided during surgery, shoulder syndrome will be greatly controlled.

The accessory nerve divides level II in the neck into two sublevels, level IIa at the front and level IIb at the back. In IIb dissection, the accessory nerve must be pulled, which causes injury to the nerve. It has been reported that the rate of IIb metastasis in early-stage oral cancer is extremely low at no more than 6% [5, 12–17]. Some clinicians therefore suggest that level IIb be exempted from neck dissection in early OSCC in order to improve HRQoL [5, 8, 17]. However, others disagree, and thus there is no consensus about the necessity of IIb dissection in T1–2N0M0 OSCC.

To assess whether IIb neck dissection should be performed in T1–T2 N0 OSCC, and its effects on OS and HRQoL, we designed this parallel-group, randomized, noninferiority trial according to the SPIRIT 2013 checklist (Additional file 1).

## Methods

### Study aims and design

Before designing this protocol, we searched the PubMed database for randomized controlled trials (RCTs) of IIb neck dissection in early-stage oral cancer on 30 May 2018, but we found no results. We repeated the search on 26 November 2018 using the keywords “IIb” and “neck” and searching only in English. We found only one RCT with a small sample size from 2018, and six prospective analyses of IIb after neck dissection from 2004 to 2018. In addition, we found two retrospective systematic reviews and meta-analyses. All of the above indicated that the rate of IIb metastasis in early-stage oral cancer is extremely low at no more than 6%. However, there is still no strong evidence to prove the necessity of IIb dissection in T1–T2N0M0 OSCC.

**Fig. 1:**
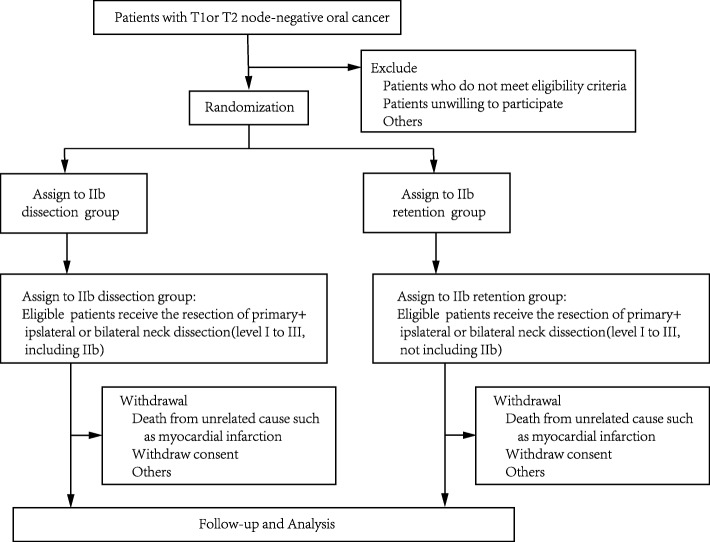
Trial profile

We are conducting a parallel-group, noninferiority randomized trial to assess whether IIb neck dissection should be performed in T1–T2 N0 OSCC and its impacts on OS and HRQoL. Our study plan is summarized in Fig. 1.

### Eligibility criteria

In this prospective, randomized, noninferiority trial, only patients who are in the clinical stages of T1–2N0M0 according to the American Joint Committee on Cancer (AJCC) Cancer staging manual, 8th edition [18], will be enrolled. Based on the National Comprehensive Cancer Networks (NCCN) guideline [19], the treatment of T1–T2 N0 oral cancer is primary resection with or without ipsilateral or bilateral cervical lymph node dissection or sentinel lymph node biopsy, and the use of radiotherapy or chemotherapy is decided according to the specific circumstances. Patients with T2+ stage oral cancer are often recommended for postoperative radiotherapy [19] and a broader range of neck dissection. Radiation may affect the sensory and motor function of the shoulder, and it has been shown that > 90% of breast cancer patients have shoulder pain and motor dysfunction after radiotherapy [20]. The probability of neck occult lymph node metastasis is greatly improved if clinical T stage is greater than 2 [17]. Therefore, we will enroll patients in stage T1 or T2. There may be discrepancies between postoperative pathological T (pT) stage and clinical T (cT) stage as some tumors can be presurgically diagnosed as T1 or T2 but have stage T3 confirmed after surgery because of the deep infiltration depth. We decided to enroll these patients. The principle for treatment of tumors located in an oral cavity site such as the soft palate, tonsil or root of the tongue differs from that for oral cancer [19]. Although it is reported that level IIb of the neck can be preserved in T1–T2 N0 oropharyngeal cancer [21, 22], such patients will not be enrolled. We will eliminate all patients in stage cN+ because the possibility of the occult metastasis in IIb increases [17]. Neck status is usually evaluated by bilateral cervical B-scan ultrasound and enhanced CT/MRI. Patients who have no suspicious lymph nodes will be enrolled after all such examinations have been conducted. In addition, patients with distant metastases will not be enrolled.

Inclusion and exclusion criteria are summarized in Table 1.

**Table 1:**
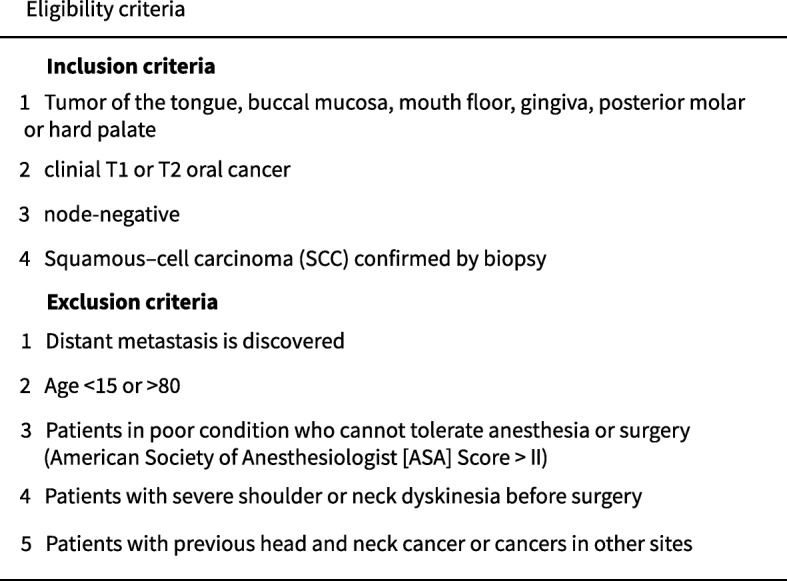
Eligibility criteria

### Interventions

After randomization, the two groups will be allocated to different interventions (see Table 2 for details). Primary resection will be 1.5–2 cm away from the tumor, and the negative margin must be obtained. If suspicious nodes in level III are found during neck dissection, and metastasis is confirmed according to the examination of frozen biopsies, we will expand neck dissection to level IV or V. For the IIb retention group, if a suspicious positive lymph node is found in level IIa during surgery and metastasis is confirmed by frozen examination, both level IIa and IIb must be dissected [5, 13].

**Table 2:**
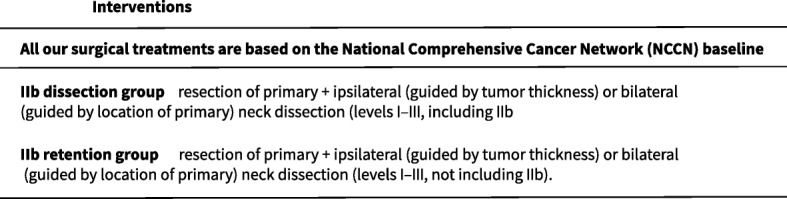
Interventions in different groups

All our surgical treatments will be based on the NCCN baseline.

### Outcomes

#### Primary outcome: overall survival (OS)

**Fig. 2:**
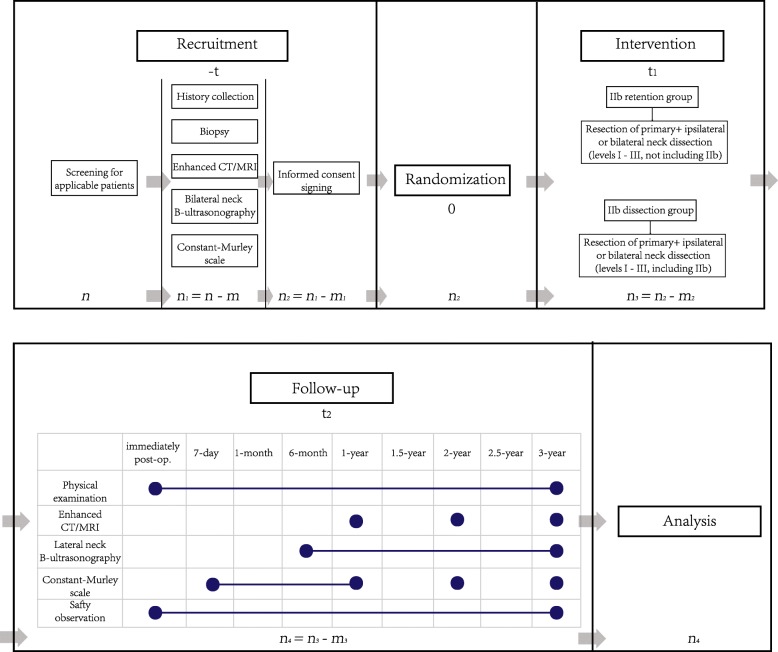
SPIRIT figure, trial visits and assessments. CT computed tomography, MRI magnetic resonance imaging

We will use the 3-year OS after surgery as the primary outcome, and follow-up at 3 months, 6 months, 1 year, 1.5 years, 2 years, 2.5 years and 3 years after surgery (Fig. 2).

#### Secondary outcomes

The secondary outcomes are HRQoL and progression-free survival (PFS). For HRQoL, we will use the Constant–Murley scale to evaluate patients’ shoulder function, with follow-up at 7 days, 21 days, 3 months, 6 months, 1 year, 2 years and 3 years after surgery.

There are many reasons for setting OS as the primary outcome. First, it is widely used as a reliable indicator for evaluating the prognosis of tumors. It is reported that the 3-year OS rate in an IIb dissection group is about 80% [1], which includes disease-free survival and living with disease. Metastasis in level IIb is extremely low, and even if it happens it is nonlethal and can be instantaneously controlled by surgery or radiotherapy. Theoretically, 3-year OS in the IIb retention group will resemble that in the dissection group. A summary of 38 RCTs [7, 23] reports no significant association between PFS duration and HRQoL. In addition, as PFS is not as reliable as OS and can also increase difficulties in follow-up, we did not use PFS as the primary outcome.

### Participant timeline

#### Recruitment

Patients will be preliminary screened at the clinic. The number of all eligible patients will be represented as *n*. After eligibility screening, we will record the number of cases that do not meet inclusion criteria as *m*, and the number of patients to be enrolled will be represented as *n*_1_ = *n* − *m*. The number of patients who are not willing to sign the informed consent form will be recorded as *m*_1_ and will be excluded. All patients (*n*_2_ = *n*_1_ − *m*_1_) who consent to participate will be randomized according to a repeatable randomized number table produced by statisticians.

#### Randomization

This is the starting point of our trial, indicating when patients officially enter the trial. Patients will be enrolled in the IIb retention or IIb dissection group according to the repeatable randomized number table.

#### Intervention

The number of patients who must be removed from our trial for any reason during intervention (surgery) will be represented as *m*_2_, and the number of patients during follow-up will be represented as *n*_3_ = *n*_2_ − *m*_2_.

#### Follow-up

**Fig. 3:**
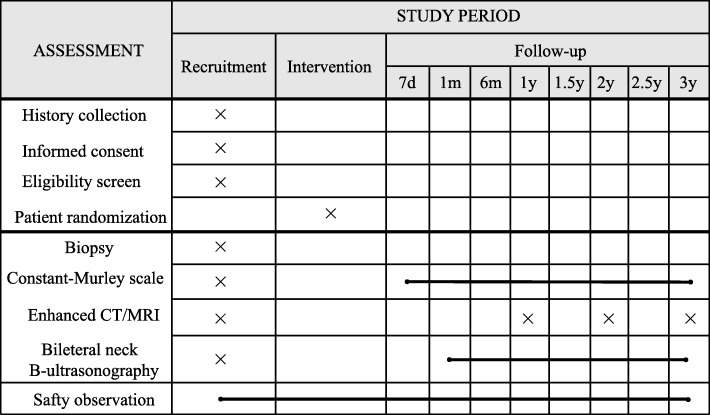
Timeline of trial. CT computed tomography, MRI magnetic resonance imaging

Follow-up timepoints include immediately, 7 days, 1 month, 6 months, 1 year, 1.5 years, 2 years, 2.5 years and 3 years after surgery. Follow-up will include physical examination, enhanced CT/MRI, bilateral neck B-scan ultrasonography, Constant–Murley score and a safety observation. There will be different evaluations at different timepoints, but overall evaluation will be the same between the two groups. The number of patients who quit our trial for any reason during follow-up will be represented as *m*_3_, and the number of patients who will be included in our analysis will be represented as *n*_4_ (see Fig. 3 for details).

### Sample size

In calculating the sample size, we assumed the 3-year OS rate in the IIb retention group will be about 78%, α = 0.05 (one-sided), power of 80% (β = 20), and that in the IIb dissection group will be 80% [1]. The noninferiority margin will be 12%, so the sample size as generated by PASS Sample Size Software 15.0 (NCSS LLC, Kaysville, Utah, USA) will be 261 for IIb retention and 260 for IIb dissection. To obtain a reasonable sample size and make sure the trial is instructive for clinical work, we combined the common opinions of oral and maxillofacial experts, and the statistician defined the noninferiority margin as 12% [24]. Although the value seems to be large, its role is to control the large sample size that would otherwise be unapproachable.

### Assignment of interventions

A statistician will write randomized code to generate a repeatable randomized number table. To reduce the predictability during enrollment, the statistician will determine block length, and a team that is not involved in our trial will keep all the blind codes safely. This team will create opaque sealed envelopes according to the randomized number table, and we will distribute patients according to this table.

### Stratification

OS can be affected by many factors, such as T stage (T1, T2), primary subsite (tongue, buccal mucosa, mouth floor, gingiva, posterior molar region or hard palate), and depth of invasion [25]. To balance the number of patients between groups and minimize the bias of the trial, we will use T stage and primary subsite for stratification.

### Blinding

Since the intervention in this clinical trial is a surgical procedure and the surgical records can be queried, surgeons and patients know the specific grouping information. After the trial we will send the data to statisticians and the evaluator who will be blinded to the groups.

### Data collection methods

#### Primary outcome

Patients will be followed-up by telephone regarding their survival status at each timepoint during the follow-up period, as shown in Fig. 2. After 3 years of follow-up of the last patient is complete, we will calculate the 3-year OS rate for both groups.

#### Secondary outcomes

For HRQoL, we will use the Constant–Murley scale to evaluate shoulder function at each follow-up timepoint. In order to improve the reliability of shoulder function evaluation, two clinicians will be systematically trained on use of the scale.

For PFS, observations will start at time of randomization and end when events (see below) occurred. In the period of time from randomization (Fig. 3) to any primary recurrence, local metastasis, distant metastasis, and other life-threatening events or death will be defined as PFS.

If a patient has not returned to the clinic for more than 2 months after the follow-up timepoint, a telephone inquiry will be conducted.

### Data management

All paper versions of the original materials will be photographed and saved in an encrypted database. All electronic data will be stored in the electronic medical records of the Shanghai Ninth People’s Hospital. All procedures for evaluating shoulder function will be filmed and saved.

### Statistical methods

#### Overall survival

This trial will be terminated when the last patient has been followed-up for 3 years. After the trial ends, the primary endpoints will be tested by means of two-sided log-rank tests.

#### HRQoL

We will use a repeated analysis of variance (ANOVA) measure to analyze changes in Constant–Murley score between the two groups. It is reported that about 67% of patients have shoulder syndrome after neck dissection even if the accessory nerve is spared [26]. Currently, two methods might work to deal with the problem of shoulder dysfunction. The first is to restore the damaged nerve with such methods as intraoperative brief electrical stimulation of the spinal accessory nerve (BEST SPIN). However, this technique has little effect [27], and literature on the treatment of damaged accessory nerves is rare. The second is retention of level IIb during surgery to preserve the integrity of accessory nerve function and structure. By measuring changes in the action potential of the accessory nerve during surgery it was found that level IIb dissection can greatly damage the accessory nerve [6]. We will use the Constant–Murley scale [28] to assess shoulder function. Although the scale’s reliability in evaluating shoulder function has been questioned [29], it has been clinically applied for more than 30 years, and it can reflect both subjective indicators (such as pain or daily activity) and objective standards (such as the muscle mobility and power). Because of the tissue defect caused by primary resection, distant free or adjacent flaps are used to restore it. In order to ensure the flaps are alive, movement control after surgery is crucial. Patients with free flaps are clinically permitted to lift the upper body on the fifth day after surgery and can also sit up in bed. On the sixth day after surgery, mild activities such as walking are permitted. Therefore, the first timepoint for evaluating shoulder function will be the seventh day after surgery, and then there will be follow-up at 1 month, 6 months, 1 year, 2 years and 3 years.

#### Progression-free survival

We will use a two-sided log-rank test to check the difference in PFS between the two groups.

#### Others

In addition, we will use post-hoc subgroup analyses on the basis of histological factors that were known to have effects on survival, such as depth of invasion of the primary tumor.

### Adverse events

Patients will be informed of all the surgical risks and adverse effects of intervention before surgery, which will be performed only if informed consent is signed. The Ethics Committee of Shanghai Ninth People’s Hospital will be notified of any adverse events (such as hemorrhagic shock, myocardial infarction, or death) that occur during surgery.

Primary recurrence or neck/distant metastasis (bone or lung) may occur in both groups. We will expand tumor resection if a primary recurrence occurs and perform radiotherapy or neck dissection depending on the tumor size. If IIb metastasis is detected during follow-up in the retention group, we will dissect level IIb and perform radiotherapy if necessary. If level IV or V is affected, we will perform additional ipsilateral or bilateral neck dissection, plus radiotherapy or chemotherapy later if needed.

## Discussion

For decades, the treatment of early-stage oral cancer has created an apparent dilemma between survival and HRQoL [1, 30–33]. To guarantee a higher survival rate, HRQoL must often be sacrificed. The neck metastatic rate in early-stage OSCC is about 30% [34], and methods of neck treatment include therapeutic dissection and observation. Data from India [1] showed that the 5-year survival rate is about 13% higher in the therapeutic group than in the control group, but overtreatment still happens in those patients whose neck lymph nodes are actually negative. As far as we know, this is also true for the treatment of breast cancer. Axillary lymph node metastasis often appears in early-stage breast cancer, at a rate of about 15–20% [35]. Previously, axillary dissection was the gold standard for treating early-stage breast cancer as it reduced recurrence and improved OS, but it also brought complications such as lymphedema, which greatly affected HRQoL for patients after surgery. Sentinel-lymph node biopsy has since replaced axillary dissection as it offers better disease control and HRQoL [36–38]. There are therefore commonalities between breast and oral cancer in their early stages.

Because there is higher occult metastasis in early-stage oral carcinoma than in early-stage breast cancer, and despite postsurgical HRQoL considerations, surgeons prefer to perform therapeutic neck dissection to enhance OS. During neck dissection in oral cancer, traction of the accessory nerve can decrease shoulder function, and damage to the nerve occurs when level IIb is dissected [26]. The occult metastatic rate for IIb is < 6% [5, 12–17], so if level IIb is retained the nerve will not be pulled and HRQoL for patients with early-stage oral cancer can be greatly increased. This trial is designed to assess whether IIb neck dissection should be performed in T1–T2 N0 OSCC, and to discuss its effects on survival rates and HRQoL.

Our trial has some limitations. First, currently it is noteworthy that, for T1 N0 OSCC, many surgeons would actually not undertake a staging neck dissection, which seems in contrast to the study. However, according to the NCCN guidelines, the recommended treatment options for T1 and T2 are the same, being resection of primary (preferred) and/or ipsilateral (guided by tumor thickness) or bilateral (guided by location of primary) neck dissection or sentinel lymph node biopsy [19]. In order to clarify the relationship between the depth of invasion and occult metastasis, we will put the depth of invasion into the final analysis. Second, at present there are many auxiliary methods for cervical examination, such as B-scan ultrasound, enhanced CT/MRI, ^18^F-fluorodeoxyglucose positron emission tomography (PET)/CT, and CT, etc. In this trial, we will use B-scan ultrasound and enhanced CT/MRI to detect occult metastatic lymph nodes. However, the sensitivity of these examinations is not high. It has been reported that PET/CT offers a distinct advantage over other conventional imaging modalities because it provides functional insights into tumor biology and tissue metabolism, and thus PET/CT has a higher sensitivity and advantages for detecting cervical metastasis. However, PET/CT also has some limitations. The cost is relatively high and, besides, the main advantage of PET/CT applies to clinically node-positive OSCC, and PET/CT has a higher false negative rate for detecting nodal involvement in the setting of a cN0 neck. NCCN guidelines currently recommend PET/CT imaging for most stage III and IV OSCC [39]. Therefore, in our trial, PET/CT is not considered for detecting neck status.

In summary, this parallel-group, randomized, noninferiority controlled trial aims to assess whether IIb neck dissection should be performed in T1–T2 N0 OSCC and to also assess the effects of IIb neck dissection on OS and HRQoL. Although there are some limitations to this study, it is still worth conducting for the advantages it may have for patients.

## Trial status

This is protocol version 2.0, 1 October 2018. Enrollment has not yet started, and is expected to be started by 1 June 2019 and be completed by 1 June 2024.

## Additional file

Additional file 1: CONSORT 2010 checklist of information to include when reporting a randomised trial. (DOC 217 kb)

## Data Availability

Not applicable.

## Abbreviations

AJCC: American Joint Committee on Cancer
BEST SPIN: Brief electrical stimulation of the spinal accessory nerve
CT: Computed tomography
HRQoL: Health-related quality of life
MRI: Magnetic resonance imaging
NCCN: National Comprehensive Cancer Networks
OS: Overall survival
OSCC: Oral squamous cell carcinoma
PET: Positron emission tomography
PFS: Progression-free survival
RCT: Randomized controlled trial

## References

[1] D'Cruz AK, Vaish R, Kapre N (2015). Elective versus therapeutic neck dissection in node-negative oral cancer. N Engl J Med.

[2] Sproll C, Freund AK, Hassel A (2017). Immunohistochemical detection of lymph node-DTCs in patients with node-negative HNSCC. Int J Cancer.

[3] de Bree R, Takes RP, Castelijns JA (2015). Advances in diagnostic modalities to detect occult lymph node metastases in head and neck squamous cell carcinoma. Head Neck.

[4] Liu KY, Durham JS, Wu J, Anderson DW, Prisman E, Poh CF (2016). Nodal disease burden for early-stage oral cancer. JAMA Otolaryngol Head Neck Surg.

[5] Santoro R, Franchi A, Gallo O, Burali G, de’ Campora E (2008). Nodal metastases at level IIb during neck dissection for head and neck cancer: clinical and pathologic evaluation. Head Neck.

[6] Lanisnik B, Zargi M, Rodi Z (2016). Electrophysiologic analysis of injury to cranial nerve XI during neck dissection. Head Neck.

[7] Kovic B, Guyatt G, Brundage M, Thabane L, Bhatnagar N, Xie F (2016). Association between progression-free survival and health-related quality of life in oncology: a systematic review protocol. BMJ Open.

[8] Celik B, Coskun H, Kumas FF (2009). Accessory nerve function after level 2b-preserving selective neck dissection. Head Neck.

[9] Taylor RJ, Chepeha JC, Teknos TN (2002). Development and validation of the neck dissection impairment index: a quality of life measure. Arch Otolaryngol Head Neck Surg.

[10] Bradley PJ, Ferlito A, Silver CE (2011). Neck treatment and shoulder morbidity: still a challenge. Head Neck.

[11] Lanisnik B (2017). Different branching patterns of the spinal accessory nerve: impact on neck dissection technique and postoperative shoulder function. Curr Opin Otolaryngol Head Neck Surg.

[12] Lea J, Bachar G, Sawka AM (2010). Metastases to level IIb in squamous cell carcinoma of the oral cavity: a systematic review and meta-analysis. Head Neck.

[13] Kou Y, Zhao T, Huang S (2017). Cervical level IIb metastases in squamous cell carcinoma of the oral cavity: a systematic review and meta-analysis. Onco Targets Ther.

[14] Villaret AB, Piazza C, Peretti G (2007). Multicentric prospective study on the prevalence of sublevel IIb metastases in head and neck cancer. Arch Otolaryngol Head Neck Surg.

[15] Haranadh S, Nandyala R, Bodagala V, Hulikal N (2018). A prospective analysis of prevalence of metastasis in levels IIB and V neck nodes in patients with operable oral squamous cell carcinoma. Oral Oncol.

[16] Ghantous Y, Akrish S, Abd-Elraziq M, El-Naaj IA (2016). Level IIB neck dissection in oral squamous cell carcinoma: science or myth?. J Craniofac Surg.

[17] Bartella AK, Kloss-Brandstatter A, Kamal M (2016). "IIb or not IIb"—the necessity of dissection in patients with oral squamous cell carcinoma. J Craniomaxillofac Surg.

[18] Lydiatt WM, Patel SG, O'Sullivan B (2017). Head and neck cancers-major changes in the American Joint Committee on cancer eighth edition cancer staging manual. CA Cancer J Clin.

[19] Colevas AD, Yom SS, Pfister DG (2018). NCCN guidelines insights: head and neck cancers, version 1.2018. J Natl Compr Cancer Netw.

[20] Johansen S, Fossa K, Nesvold IL, Malinen E, Fossa SD (2014). Arm and shoulder morbidity following surgery and radiotherapy for breast cancer. Acta Oncol.

[21] Gross BC, Olsen SM, Lewis JE (2013). Level IIB lymph node metastasis in oropharyngeal squamous cell carcinoma. Laryngoscope.

[22] Koybasioglu A, Bora Tokcaer A, Inal E, Uslu S, Kocak T, Ural A (2006). Accessory nerve function in lateral selective neck dissection with undissected level IIb. ORL J Otorhinolaryngol Relat Spec.

[23] Kovic Bruno, Jin Xuejing, Kennedy Sean Alexander, Hylands Mathieu, Pedziwiatr Michal, Kuriyama Akira, Gomaa Huda, Lee Yung, Katsura Morihiro, Tada Masafumi, Hong Brian Y., Cho Sung Min, Hong Patrick Jiho, Yu Ashley M., Sivji Yasmin, Toma Augustin, Xie Li, Tsoi Ludwig, Waligora Marcin, Prasad Manya, Bhatnagar Neera, Thabane Lehana, Brundage Michael, Guyatt Gordon, Xie Feng (2018). Evaluating Progression-Free Survival as a Surrogate Outcome for Health-Related Quality of Life in Oncology. JAMA Internal Medicine.

[24] Althunian TA, de Boer A, Klungel OH, Insani WN, Groenwold RH (2017). Methods of defining the non-inferiority margin in randomized, double-blind controlled trials: a systematic review. Trials.

[25] Sowder JC, Cannon RB, Buchmann LO (2017). Treatment-related determinants of survival in early-stage (T1-2N0M0) oral cavity cancer: a population-based study. Head Neck.

[26] van Wilgen CP, Dijkstra PU, van der Laan BF, Plukker JT, Roodenburg JL (2004). Shoulder complaints after nerve sparing neck dissections. Int J Oral Maxillofac Surg.

[27] Barber B, Seikaly H, Ming Chan K (2018). Intraoperative brief electrical stimulation of the spinal accessory nerve (BEST SPIN) for prevention of shoulder dysfunction after oncologic neck dissection: a double-blinded, randomized controlled trial. J Otolaryngol Head Neck Surg.

[28] Constant CR, Murley AH (1987). A clinical method of functional assessment of the shoulder. Clin Orthop Relat Res.

[29] Vrotsou K, Avila M, Machon M (2018). Constant-Murley Score: systematic review and standardized evaluation in different shoulder pathologies. Qual Life Res.

[30] Huang SF, Kang CJ, Lin CY (2008). Neck treatment of patients with early stage oral tongue cancer: comparison between observation, supraomohyoid dissection, and extended dissection. Cancer.

[31] Kelner N, Vartanian JG, Pinto CA, Coutinho-Camillo CM, Kowalski LP (2014). Does elective neck dissection in T1/T2 carcinoma of the oral tongue and floor of the mouth influence recurrence and survival rates?. Br J Oral Maxillofac Surg.

[32] de Bree R, van den Brekel MWM (2015). Elective neck dissection versus observation in the clinically node negative neck in early oral cancer: do we have the answer yet?. Oral Oncol.

[33] Bluemel C, Rubello D, Colletti PM, de Bree R, Herrmann K (2015). Sentinel lymph node biopsy in oral and oropharyngeal squamous cell carcinoma: current status and unresolved challenges. Eur J Nucl Med Mol Imaging.

[34] Samant S (2014). Sentinel node biopsy as an alternative to elective neck dissection for staging of early oral carcinoma. Head Neck.

[35] Bundred NJ, Barnes NL, Rutgers E, Donker M (2015). Is axillary lymph node clearance required in node-positive breast cancer?. Nat Rev Clin Oncol.

[36] Mamounas EP, Kuehn T, Rutgers EJT, von Minckwitz G. Current approach of the axilla in patients with early-stage breast cancer. Lancet. 2017. ISSN 0140-6736. 10.1016/S0140-6736(17)31451-4 28818521

[37] Pesce C, Morrow M (2013). The need for lymph node dissection in nonmetastatic breast cancer. Annu Rev Med.

[38] Kobayashi R, Shiraishi K, Iwase S, Ohtomo K, Nakagawa K (2015). Omission of axillary lymph node dissection for clinically node negative early-stage breast cancer patients. Breast Cancer.

[39] Wright CL, Washington IR, Bhatt AD, Knopp MV (2019). Emerging opportunities for digital PET/CT to advance locoregional therapy in head and neck cancer. Semin Radiat Oncol.

